# Consultants and General Practitioners

**Published:** 1893-11-04

**Authors:** 


### The Hospital

AN INSTITUTIONAL JOURNAL OF

Science, Medicine, Hygiene, Nursing, and News.

*For Hospitals, Asylums, Infirmaries, Dispensaries, Medical Practitioners, Students, and Nurses.*

VOL. XV.—No. 371.] [NOV. 4, 1893.

### CONSULTANTS AND GENERAL PRACTITIONERS.

THE *Times* has opened its columns for a discussion on the question of the relations between medical consultants and general practitioners of the venerable art. This is a sure indication that interest in the subject is no longer confined to the ranks of medical men, but has extended to the whole reading community. One of the *Times'* correspondents, who signs himself "F.R.C.P.," affirms that, in this country, neither in London nor in the provinces is there any such person as a "pure consultant" known. There are, he maintains, no pure consultants, but there ought to be. That is really the gist of the whole question. There is a class of "approximate consultants;" and it has come to be universally felt that such a class is both useful and necessary. But, argues "F.R.C.P.," the class which is only approximately a consulting class ought to devote itself entirely to consultation work; and that would bring up the art of medicine and its professors to the highest possible standard of general usefulness, and at the same time satisfy the craving which demands obedience to the obvious fitness of things.

There are several points here to be considered. First, we may ask, is it actually the case that we have not a single pure consultant in the whole profession of medicine? The answer must be that in plain truth we have not. But what does this lead to in the ranks of "approximate consultants," as they are called? It leads to immeasurable confusion; and, among other things, to the painful degradation of those tried and proved seniors who are and who deserve to be the most trusted members of our profession. One fact in this connection, which "F.R.C.P." points out, is almost sublime in its absurdity. He tells us, and we all know it quite well, that when a patient consults the President of the Royal College of Physicians a fee of two guineas is demanded of him. If the same patient on the same day were to consult Dr. Fledgeling, M.R.C.P., ætat. 27, the newly-appointed physician to out-patients at the suburban hospital of Cockney-externa, he would be charged exactly two guineas. That is to say, according to the arrangements which now prevail in the higher ranks of medicine, the pay of a Lieutenant fresh from Sandhurst is and ought to be on exactly the same scale as the pay of the Commander-in-Chief of all her Majesty's forces. If this is not sublime in its absurdity we would like to know what, in the estimation of medical men, would be considered sublime! Surely it is to inflict a degradation, and that of the most painfully practical kind, upon every senior who is thus compelled, whether he will or not, to place himself in competition and on a level with the rawest graduate of the schools.

But it is the general practitioner who thinks he has the most urgent reasons for lifting up his voice against the present anomalous, and, in many respects, absurd condition of things. On the one hand, he says, there is a class who call themselves "consultants," and who claim to possess more knowledge and consequently to exact higher fees than I do. These swoop down like hawks upon my flocks of pigeons and carry away the fattest, in spite of all I can do, without leaving me so much as a single tail feather. On the other hand are the hospitals, spacious, comfortable, inviting; with the best of doctors, the kindest and most skilful of nurses, and everything that money can buy in the way of food and physic. These throw their doors wide open for all the hungry and discontented sparrows among my patients to fly in without let or hindrance. The family practitioner is thus ground between the upper and the nether millstone; and which of them is the harder he finds it impossible to tell. What he does feel is that if he could have his way he would change places for a time, first with the "approximate consultant" and then with the hospital, and ask them from his new position how they liked the change. It cannot be denied that between the approximate consultant who takes the cream of general practice when he can get it, and the hospital which swallows up the butter milk, the general practitioner often has a very hard time of it indeed. It speaks volumes for his good sense and kindness of heart that he manages, in spite of the grindings and robberies of which he is the subject, to keep on excellent terms with both.

But now, is this to go on for ever? Is there never to be a change? Are all classes of medical men so intractable and so unbusiness-like that, though they can see and wring their hands about the confusion for decade after decade, yet they never succeed in evolving any order out of the miserable and discreditable chaos? Surely the time has come for the establishment of a new order of things! "F.R.C.P." indeed intimates as much; and he calls upon the Royal College of Physicians to give facilities for the founding of a class of really pure consultants analogous to the order of Q.C.'s in the law. Is this a practical suggestion? It most certainly ought to be, but to effect even this small advancement the Fellows must be up and doing. The College of Physicians, even under its present distinguished head, has not proved itself a body capable of statesmanship. It is a great pity; but it is true. What is the reason we cannot tell; but whilst the College of Physicians has among its members individual practitioners of the most brilliant and statesman-like capacity, it seldom succeeds in doing anything as a body, which affords practical evidence of their existence. This, we repeat, is a pity, and still more a pity. There is not much hope, therefore, in the College of Physicians.

Where then are we to look for the beginnings of better things? We must look to practical and spontaneous evolution, to individual initiative, that is to say. The question lies in a nutshell, and it is, like so many other questions, a question of money. Some man, who values the honour of his profession and his own improvement in knowledge and mental capacity more than money, must at a given stage of his career stand for higher things. Let us suppose he is 55, or even 60 years of age, and has provided a modest competency against old age of twenty-five or thirty thousand pounds, or even less. He can now afford to run a little risk. Let him run the risk. Instead of seeing all and sundry who come to him for the uniform fee of two guineas, let him charge, say, ten guineas for a single consultation in his own room, and twenty, fifty, or a hundred guineas for a series of such consultations. But they must of course be real consultations, that is, consultations with the family physician already in charge. No doubt there would be some loss of money to such a consultant at the outset. But look at the gain in prestige, in leisure, in mental freshness and capacity, in the power of keeping up with every new development in medical science and art! Moreover, a consultant of this class who should secure the confidence of his professional brethren would, in the long run, reap higher rewards even in money than those who continued to practise in the old chaotic and unsatisfactory way. The gain to the individual consultant would be great; the gain to practical medicine would be enormous. The clinical literature of the period would acquire a breadth, a philosophic depth and insight, and a practical utility to which no previous clinical literature has ever approached.

Of course such a development would lead to the hopeless disestablishment and disendowment of whole regiments of so-called consultants; and an admirable result it would be. We are all educated practitioners of medicine. There needs no such classification as that which places some amongst general practitioners and others amongst physicians. The practitioner who practises in the family is the family physician; the specialist is neither physician nor surgeon, but aurist, oculist, or dentist, as the case may be. The real consultant, when he is once created, or rather when he evolves himself, as he will do sooner or later, and possibly from the ranks of family practice, will take his place as the trusted head of his brethren by virtue, not of college edicts or State regulation, but of proved and cheerfully recognised capacity. The new order of real consultants when it is established will be open to the whole profession, and will consist entirely of those who have the capacity, the courage, and the material resources to justify them in opening its doors and taking their place in its ranks.

